# Basic Overview of Conventional Coronary Angiography for Planning Cardiac Surgery

**DOI:** 10.7759/cureus.52942

**Published:** 2024-01-25

**Authors:** Emeka B Kesieme, Christopher O Iruolagbe, Benjamin I Omoregbee, Ismail M Inuwa

**Affiliations:** 1 Cardiothoracic Surgery, Irrua Specialist Teaching Hospital, Irrua, NGA; 2 Cardiothoracic Surgery, Castle Hill Hospital, Cottingham, GBR; 3 Cardiology, Rosalind Franklin University of Medicine and Science/Chicago Medical School, Chicago, USA; 4 Cardiothoracic Surgery, Hull University Teaching Hospitals NHS Trust, Hull, GBR; 5 Cardiothoracic Surgery, Aminu Kano Teaching Hospital, Kano, NGA

**Keywords:** cardiac catheterization, coronary anatomy, aortography, ventriculography, coronary angiography

## Abstract

Coronary angiography is a common procedure performed by the cardiologist to evaluate coronary atherosclerotic disease (CAD) and the result is utilized by both cardiologists and cardiac surgeons to perform catheter and surgical interventions on the coronary artery. In addition to evaluating CAD, other useful investigative modalities such as left ventriculography and aortography can be performed at the time of coronary angiography. Despite its limitations and the emergence of newer investigative modalities like coronary computed tomography angiography, intravascular ultrasound scan, and magnetic resonance coronary angiography, conventional coronary angiography has remained the gold standard for the evaluation of coronary artery disease. Hence, it remains an investigative modality that every member of the cardiothoracic team performing coronary artery bypass grafting must learn how to interpret.

## Introduction and background

Coronary angiography has remained the gold standard in evaluating coronary atherosclerotic disease (CAD) since its inception in 1958 by Dr. Mason Sones [1]. It gives an idea of the location and severity of the diseased coronary vessel and provides information on the extent of collateral circulation. However, it is invasive and may be associated with complications [2].

Morphological evaluation of the degree of stenosis is usually not the only criterion for coronary revascularization as suspicious and high-grade coronary artery stenoses do not necessarily induce relevant myocardial ischemia in the territory supplied by the diseased coronary artery. Hence, it may be necessary to perform intracoronary pressure wire studies and measure fractional flow reserve (FFR) or instantaneous wave-free ratio (iFR) during coronary angiography in the presence of coronary stenosis.

In the presence of hemodynamically significant coronary stenosis, revascularization can be performed in the form of percutaneous coronary intervention at the same time as performing coronary angiography or by coronary artery bypass grafting.

Intravascular ultrasound (IVUS) may be useful to help define coronary lesions anatomically when limitations may occur in the angiographic examination due to overlapping of coronary vessels, assessment of ambiguous anatomy as in intermediate severity of stenosis, and measuring the dimensions of a lesion [3].

In this review, we have discussed the basic views of coronary angiography and a simplified step-by-step method of interpreting coronary angiograms relevant to cardiac surgery. The usefulness of left ventriculography and aortography has been discussed.

## Review

Methods

A literature review of coronary angiography was done from 1970 to date using a manual library search, journal publications on the subject, and MEDLINE (Medical Literature Analysis and Retrieval System Online) database search. Full texts of the materials, including those of relevant references, were collected and studied. Information relating to angiographic views, interpretation, left ventriculography, aortography, and complications were extracted from the materials.

Results

Access for the Procedure and Commonly Used Catheters

Access for coronary angiography is mostly through the femoral artery and the radial artery. The brachial artery is rarely used. The most commonly used catheters include the Judkins catheter and the Amplatz catheter. For catheterization of the left coronary artery, a 4mm Judkins catheter (JL4) is often used, and a 4.5mm or 5mm Judkins catheter is used in patients with dilated ascending aorta. For catheterization of the right coronary artery, the 4.0mm Judkins catheter (JR4) is used [4]. The right Judkins catheter (JR) is also used for performing coronary angiograms to evaluate saphenous vein grafts in post-coronary artery bypass and left internal mammary (LIMA) graft [5].

Common Views for Coronary Angiography

Right anterior oblique (RAO) view: The image intensifier is placed on the right side of the patient. This view is recognized by the presence of the spine on the left of the screen and the ribs which appear to descend to the right side of the image [3,6].

Left anterior oblique (LAO) view: The image intensifier is placed on the left side of the patient. The LAO view is recognized by the presence of the spine on the right of the screen and the ribs, which appear to descend to the left side of the image [3,6].

Anteroposterior view: The image intensifier is directly over the patient with the beam traveling perpendicularly back to front [3,6].

Caudal view: The image intensifier is tilted towards the feet of the patient and the diaphragm is usually not seen in the image. It is the best view for the left anterior descending artery (LAD) and its diagonal branches [3,6].

Cranial view: The image intensifier is tilted towards the feet, and the diaphragm is usually seen over the cardiac silhouette. This view displays the circumflex artery and the left main coronary artery (LMCA) [3,6].

Recognition of Coronary Arteries

The LAD: The LAD descends towards the apex of the heart. The septal perforators come off the LAD at a right angle to the vessel and run toward the right ventricle whereas the diagonal branches course toward the circumflex artery [3]. The LAD splits near the apex like a mustache or the bifid tail of a whale, and this is referred to as the mustache sign or whale tail sign [7]. The circumflex artery is close to the thoracic spine in all views of the LAD [3]. The obtuse marginal (OM) branches come off the circumflex artery and run toward the LAD territory [3].

Left Coronary Angiogram

**Figure 1:**
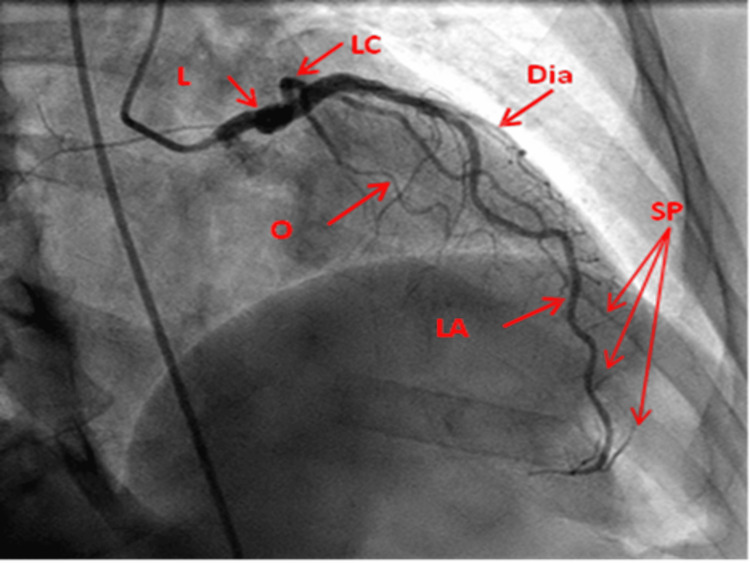
Right anterior oblique cranial view of the left coronary artery. L: Left main coronary artery; LA: Left anterior descending artery; LC: Left circumflex artery; Dia: Diagonal artery; O: Obtuse marginal; SP: Septal perforators Reproduced with permission from Sachdev et al. [8]. Copyright 2018 Cardiofellow Newsletter

RAO cranial view: This view displays the LMCA clearly. Beyond the proximal aspect, the LAD and circumflex artery are overlapped. Hence, this is not a reliable view for these vessels at this level. It, however, displays the diagonal branches on the left and septal perforators just opposite at a right angle, and the mid and distal LAD (Figure 1).

**Figure 2:**
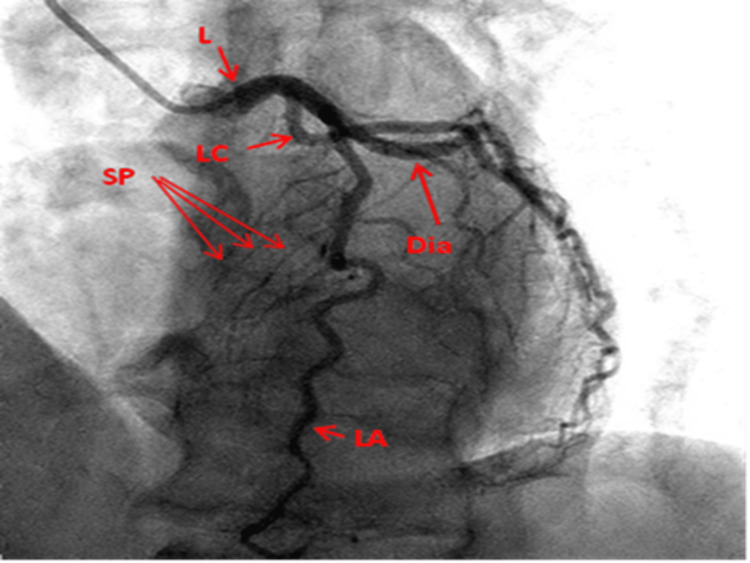
Left anterior oblique cranial view of the left coronary artery. L: Left main coronary artery; LA: Left anterior descending artery; LC: Left circumflex artery; Dia: Diagonal artery; O: Obtuse marginal; SP: Septal perforators Reproduced with permission from Sachdev et al. [8]. Copyright 2018 Cardiofellow Newsletter

LAO cranial view: This view displays the LMCA and the LAD parallel to the spine giving off the diagonal to the right and septal branches to the left while the circumflex artery courses towards the right. Stenosis at the ostia of diagonal branches is easily seen. It provides an excellent view in left dominant circulation as the posterior descending artery (PDA) branches are clearly visualized (Figure 2).

**Figure 3:**
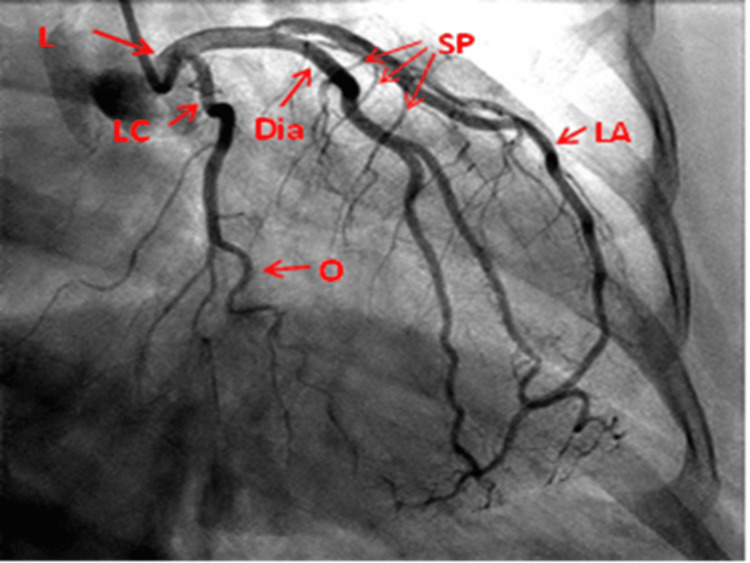
Right anterior oblique caudal view of the left coronary artery. L: Left main coronary artery; LA: Left anterior descending artery; LC: Left circumflex artery; Dia: Diagonal artery; O: Obtuse marginal; SP: Septal perforators Reproduced with permission from Sachdev et al. [8]. Copyright 2018 Cardiofellow Newsletter

RAO caudal view: This view clearly displays the origin/stem of the entire circumflex artery and its OM branches especially lesions at the ostium of the OM branches and ramus intermedius when present. It also displays the distal LMCA and proximal LAD. Beyond this point, overlapped diagonal branches may obscure the LAD (Figure 3).

**Figure 4:**
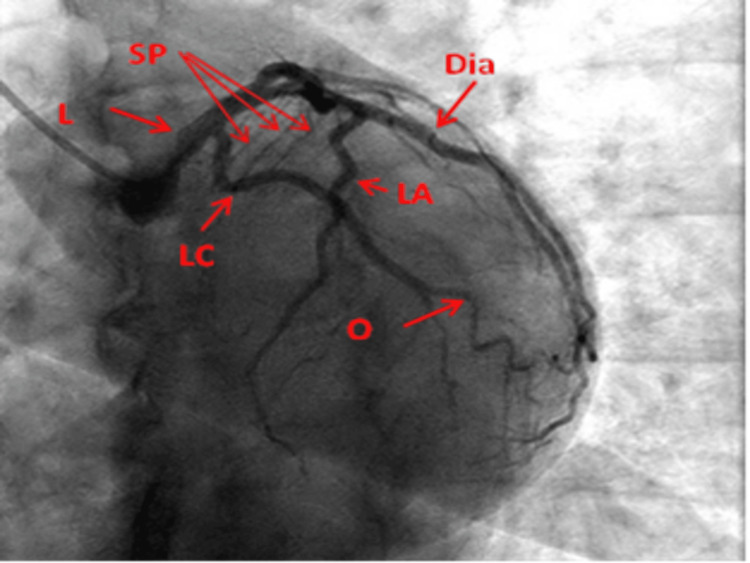
Anteroposterior caudal view of the left coronary artery. L: Left main coronary artery; LA: Left anterior descending artery; LC: Left circumflex artery; Dia: Diagonal artery; O: Obtuse marginal; SP: Septal perforators Reproduced with permission from Sachdev et al. [8]. Copyright 2018 Cardiofellow Newsletter

AP caudal view: This view displays the midsegment of the LAD and the distal LAD, its diagonal branches, and their ostia. The distal LMCA, proximal LAD, circumflex branch, and its OM branches may not be well displayed in this view (Figure 4).

**Figure 5:**
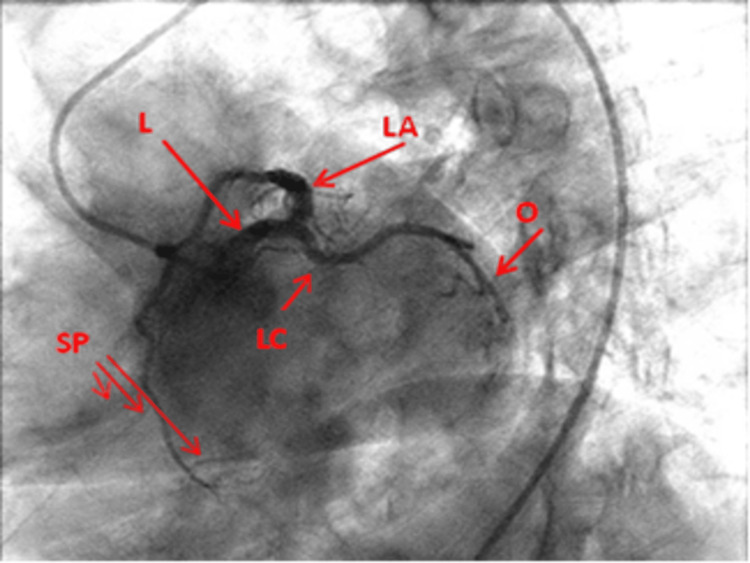
Left anterior oblique caudal/spider view of the left coronary artery. L: Left main coronary artery; LA: Left anterior descending artery; LC: Left circumflex artery; Dia: Diagonal artery; O: Obtuse marginal; SP: Septal perforators Reproduced with permission from Sachdev et al. [8]. Copyright 2018 Cardiofellow Newsletter

LAO caudal/spider view: This view displays the left mainstem coronary artery and its bifurcation into proximal LAD, proximal and midsegment of the circumflex artery, and ramus intermedius if present (Figure 5).

Right Coronary Angiogram

**Figure 6:**
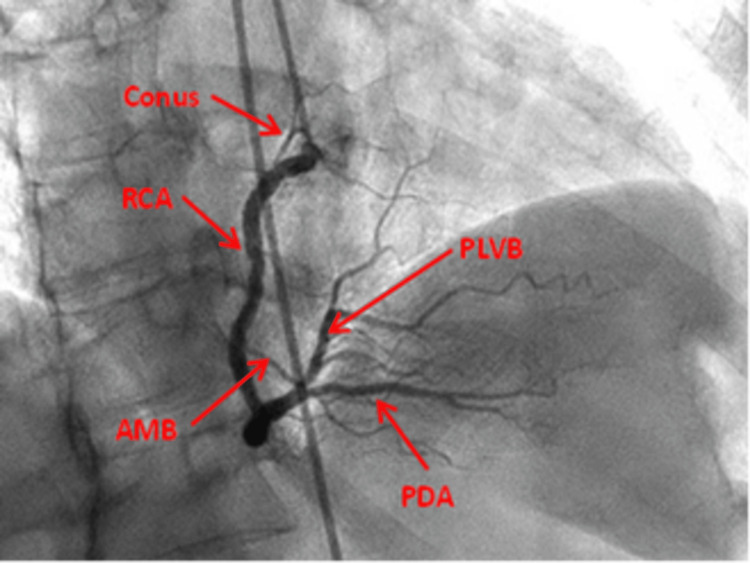
Right anterior oblique straight view of the right coronary artery. RCA: Right coronary artery; AMB: Acute marginal branch; PDA: Posterior descending artery; PLVB: Posterior left ventricular branch Reproduced with permission from Sachdev et al. [8]. Copyright 2018 Cardiofellow Newsletter

RAO view: This view displays the ostia of the right coronary artery (RCA), the proximal RCA, and the mid-RCA. Depending on the angle, it may reveal the more proximal and clearer division of RCA, hence the ostial lesions of the terminal branches may be obvious and, in some angles, overlapping of branches may be seen (Figure 6).

**Figure 7:**
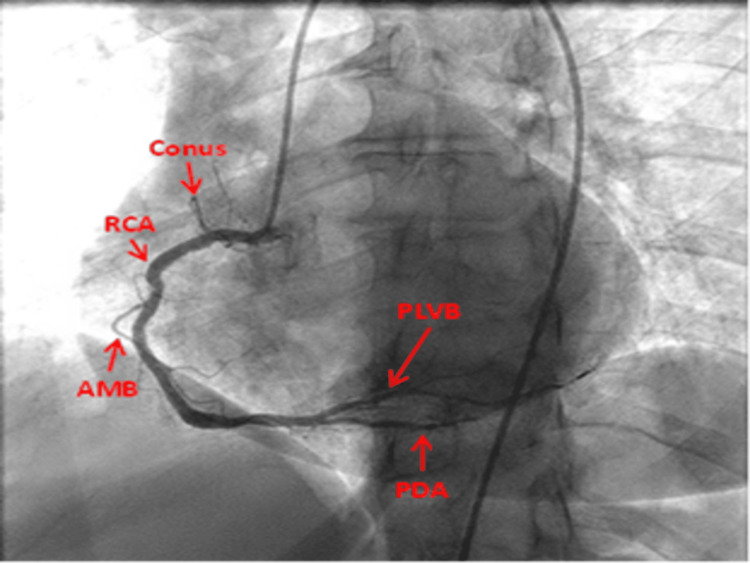
Left anterior oblique straight view of right coronary artery. RCA: Right coronary artery; AMB: Acute marginal branch; PDA: Posterior descending artery; PLVB: Posterior left ventricular branch Reproduced with permission from Sachdev et al. [8]. Copyright 2018 Cardiofellow Newsletter

**Figure 8:**
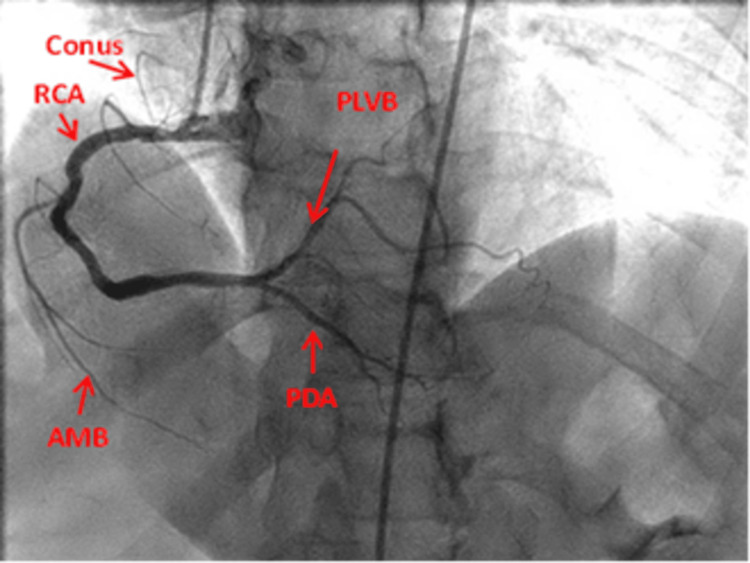
Anteroposterior-Left anterior oblique cranial view of the right coronary artery. RCA: Right coronary artery; AMB: Acute marginal branch; PDA: Posterior descending artery; PLVB: Posterior left ventricular branch Reproduced with permission from Sachdev et al. [8]. Copyright 2018 Cardiofellow Newsletter

LAO view: This view shows the ostia of RCA, the proximal and the mid-RCA, and the body of RCA as a C-shape vessel. The PA cranial reveals the distal RCA and its bifurcation into the PDA and the posterior left ventricular branches (PLVB) (Figures 7, 8).

Interpretation of a Coronary Angiogram

Assessment of a lesion should be performed in at least two views because the coronary angiogram provides a two-dimensional (2D) image of a three-dimensional (3D) structure.

Coronary dominance: In right dominant circulation, the RCA gives off the PDA. In left dominant circulation, the circumflex artery gives off the PDA, and in codominant circulation, the PDA comes off both the RCA and the circumflex artery. In patients undergoing elective surgical revascularization, left coronary dominance has been found to be associated with an increased risk of major adverse cardiac and cerebrovascular events in the long term [9].

Location of stenosis: CAD may affect any or all the branches of the coronary artery (single-vessel disease, two-vessel disease, or three-vessel disease). It may also affect the proximal, mid, or distal aspect of the coronary artery. It can be ostial (within 3mm of the origin of the vessel) or non-ostial.

Length and diffuse nature of stenosis: The length could be 1cm, 1-2cm, or ≥2cm. The length of stenosis is physiologically important as a long stenotic lesion may result in more ischemia than a focal severe lesion [10]. Stenosis may be single or may involve a long segment of the coronary artery (diffuse disease). Diffuse disease may be tandem or sequential. Tandem lesions can be defined as multiple stenotic lesions in series with an intervening normal segment of the coronary artery. The term 'sequential' is preferred for longer normal segments between stenosis [11]. CAD with diffuse long segments is often challenging to treat. Revascularization with PCI has necessitated the use of multiple stents or very long stents, and the procedure is often complicated by a high risk of stenosis and restenosis. The outcome has been improved with Resolute™ zotarolimus-eluting stent (Medtronic, Dublin, Ireland) [12]. An angiographic lesion longer than 2cm contributes to the increased complexity of CAD. This factor in addition to the patient's comorbidities and some procedural factors requires heart team discussion regarding the best modality for revascularization [10].

Morphology of stenosis: Lesion may be concentric, eccentric, or have excessive tortuosity The contour of stenosis may be smooth, irregular, or ulcerated.

Size: The size of the coronary arteries is not usually included in the angiography result; however, surgeons have often visually estimated the size of the coronary arteries using this study. Studies have revealed that the mean diameter of major coronary arteries can be determined angiographically [13]. Although it is technically challenging to anastomose diseased small coronary arteries, Ngaage et al., in their analysis of coronary anastomoses of 5171 patients, supported undertaking the challenging task of constructing anastomoses in these vessels when technically feasible as this did not significantly increase the risk of early postoperative major adverse cardiac events [14].

Severity of stenosis: Coronary artery stenosis can be classified as minimal (50%), moderate (50-70%), and severe (≥70%). Total occlusion is complete (100%). Chronic total occlusion is defined as a documented duration of occlusion of at least three months with absolutely no flow through the stenotic lesion. Recent occlusions are those of one to three months duration while subacute occlusions occur within four weeks after an acute myocardial infarction [15,16].

The cross-sectional area of stenosis is greater than the luminal or diameter stenosis because the lumen of the vessel is more often eccentric, not circular, which is the assumption on which the estimates of luminal diameter stenosis are made [6]. Hence, 50% diameter or luminal stenosis corresponds to 75% cross-sectional area stenosis, 75% diameter or luminal stenosis corresponds to 95% cross-sectional area stenosis, while 90% diameter or luminal stenosis corresponds to 99% cross-sectional area stenosis [15].

The extent of luminal or diameter stenosis does not always correlate with the hemodynamic significance of the lesion. FFR and iFR are the two most used physiological methods of identifying hemodynamically significant lesions. FFR is defined as the ratio of the distal coronary pressure (pressure distal to the stenosis) and pressure in the aorta after induced maximal hyperemia and across the entire cardiac cycle [16]. iFR measures the resting pressure gradient across a coronary lesion during mid‐diastole without the need to administer a hyperaemic agent unlike in FFR [17]. Revascularization is indicated if the iFR ≤ 0.89 or the FFR ≤ 0.80. These thresholds indicate the presence of hemodynamically important stenosis. In the FAME (Fractional Flow Reserve Versus Angiography for Multivessel Evaluation) 1 trial, the primary endpoint (composite of death, myocardial infarction, and repeat revascularization) was significantly reduced in patients with FFR-guided revascularization (FFR ≥ 0.8) than those with standard angiographically guided revascularization [18] During angiography, thrombolysis in myocardial infarction (TIMI) flow rate is used to evaluate the flow rate of contrast material in the coronary vessel. Four grades have been defined. In TIMI 0, there is no antegrade flow beyond the stenosis. In TIMI 1, there is a faint antegrade flow of contrast beyond the stenotic area, but contrast fails to opacify the entire coronary bed distal to the occlusion while in TIMI 2, there is a delayed antegrade flow across the stenotic area but with complete filling of the vessel distal to stenosis. TIMI 3 is characterized by normal flow with complete filling distally [19].

Thrombus: The presence of a thrombus within the coronary artery is suspected if there is a discrete intraluminal defect with defined borders and separated from the adjacent wall. They are more likely to be seen in patients with acute coronary syndrome [5].

Calcification: Coronary artery calcification can be classified as none/mild, moderate, and severe. In severe calcification, radiopacity is seen without cardiac motion before contrast injection and usually affects both sides of the arterial lumen. In moderate calcification, radiopacity is noted only during the cardiac cycle before contrast injection [20]. Significant coronary artery calcification poses significant technical challenges both during percutaneous coronary intervention and coronary artery bypass grafting (CABG). Performing a distal anastomosis may become a nightmare because of difficulty in passing the needle through a calcified arterial wall, and even when this is possible, the calcified arterial wall is non-compliant and its recoil may narrow the anastomosis [21]. Adjunct coronary endarterectomy, a procedure in which complete intima and media containing calcification and plaques are extracted, can be performed in conjunction with CABG to ensure optimal and complete myocardial revascularization in these patients with extensive wall calcification or atherosclerotic changes [22]. Coronary endarterectomy is mainly performed in the RCA and LAD. In patients with aortic stenosis, or mitral stenosis, annular calcification may be obvious on coronary angiogram. Mitral valve calcification is characteristically crescent-shaped [5].

Myocardial bridging: In myocardial bridging, a segment of the epicardial coronary artery (tunneled artery) traverses through the myocardium (myocardial bridge) for a portion of its length. Angiographically, coronary narrowing apparently occurs in the tunneled artery during systole while in diastole, the vessels return to their normal caliber [23]. This is most common in LAD. Exposing the coronary artery for grafting may become technically challenging in patients with myocardial bridging.

Presence of collaterals: Collaterals may be seen on angiography. The presence of collateral circulation is of important clinical significance in occlusive coronary artery disease, as it provides an alternative source of blood supply to the myocardium and preserves myocardial function [24]. The extent of myocardial necrosis and the prevalence of aneurysm formation have been shown to be smaller in patients with angiographically confirmed collateral vessels compared to patients without collateral supply [25,26]. In 20-25% of normal individuals, there are preformed collateral arteries preventing myocardial ischemia during brief vascular occlusion whereas in those with chronically diseased coronaries, collateral arteries preventing myocardial ischemia during a brief occlusion are present in every third individual [27]. After revascularization, collaterals tend to regress immediately and regression extends for many months after the procedure. The implication is that acute coronary syndrome is likely to occur following an acute reocclusion as collaterals would not have been instantaneously recruited [28].

Abnormal coronary artery: Ectopic origin of coronary artery or branches of the coronary artery, and coronary artery abnormalities may be visualized in coronary angiography.

Left Ventriculography

Left ventriculography has been performed in addition, in some patients undergoing coronary angiography; however, its use is gradually being replaced by echocardiography, cardiac MRI, nuclear MRI, cardiac computed tomographic angiography (CCTA), and radionucleotide ventriculography [4,23].

**Figure 9:**
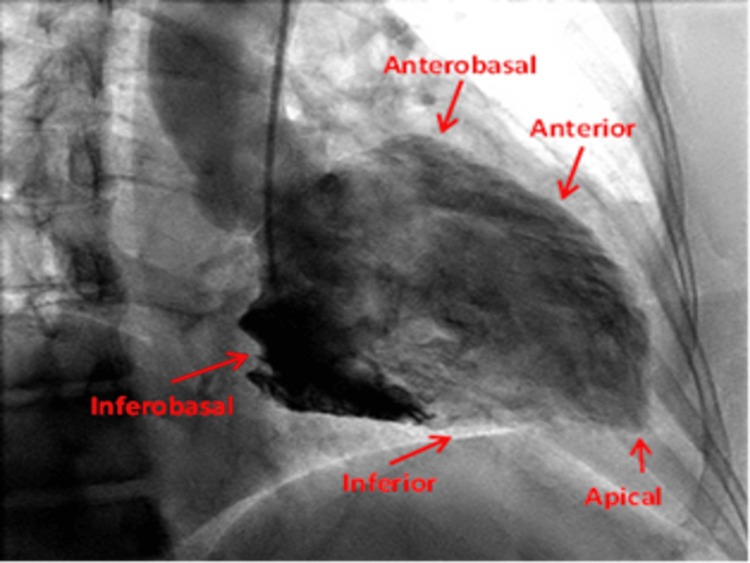
Right anterior oblique/Cranial view displaying the anterior, anterobasal, apical, inferior, and inferobasal wall. Reproduced with permission from Sachdev et al [8]. Copyright 2018 Cardiofellow Newsletter

**Figure 10:**
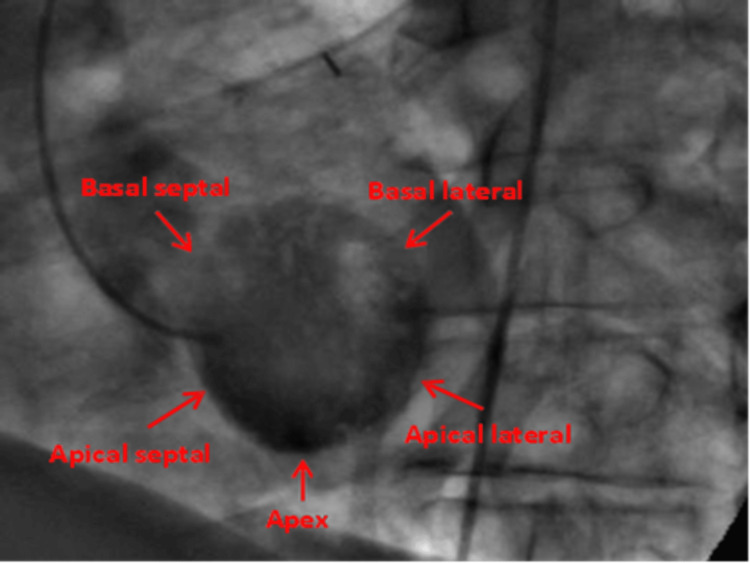
Left anterior oblique/Cranial view displays the lateral, septal, and apical walls. Reproduced with permission from Sachdev et al. [8]. Copyright 2018 Cardiofellow Newsletter

RAO projection is the most commonly performed view and the gold standard and it displays the anterior, anterobasal, apical, inferior, and inferobasal walls [4,23]. LAO view is only necessary in evaluating the posterolateral wall. The LAO view displays the lateral, septal, inferior, and apical walls. [4,23] (Figures 9, 10).

Left ventriculogram provides information about the ejection fraction of the left ventricle, left ventricular volume, detection of regional wall motion abnormalities, presence of ventricular thrombus, mitral regurgitation, presence of aneurysm, pseudoaneurysm and ventricular septal defect [5]. The regional wall motion can be normal, hypokinetic, kinetic, or dyskinetic.

Outpouching of the wall with a broad-based neck exhibiting akinetic or dyskinetic movement suggests true aneurysm while those with narrow neck suggests pseudoaneurysm or contained rupture [29]. Ventricular thrombus is suggested by the presence of an apical filling defect [29].

Mitral valve regurgitation can be evaluated by angiography. Angiographic evaluation of the severity of mitral regurgitation is based on the ejection of contrast media into the left atrium through the insufficient mitral valve [30].

Angiographic grading of mitral regurgitation [30-32] includes: (i) Grade 1+ (mild): Regurgitation essentially clears with each beat and never opacifies the entire left atrium; (ii) Grade 2+ (moderate): Regurgitation does not clear with one beat and opacifies the entire left atrium after several beats; (iii) Grade 3+ (moderately severe): The left atrium is opacified completely and achieves equal opacification to the left ventricle; (iv) Grade 4+ (severe): The entire left atrium is opacified within one beat and becomes denser with each beat, with associated refluxing into the pulmonary veins during systole.

Aortography

This has been largely overtaken by newer investigative modalities like echocardiography, CT scan, and MRI; however, it remains useful in the assessment of the dimension of the aortic root and the ascending aorta, severity of aortic regurgitation, localization of coarctation of the aorta and establishing the origin and patency of coronary artery grafts [3].

Angiographic grading of severity aortic regurgitation is based on the injection of contrast into the left ventricle through the insufficient aortic valve [29,33]. The grading is as follows [32,33]: (i) Mild (1+): A little contrast enters the left ventricle during diastole and clears with each systole; (ii) Moderate AR (2+): Contrast enters the left ventricle with each diastole, but the left ventricle is less dense than the aorta; (iii) Moderately severe AR (3+): The left ventricle has the same density as the ascending aorta; (iv) Severe AR (4+) - Dense complete, opacification of the left ventricle occurs on the first beat; it is more densely opacified than the ascending aorta.

Complications of Coronary Angiography

The risk of major complications following coronary angiography occurs in less than 2% of cases [2]. These include local vascular injury (hematoma, pseudoaneurysm, retroperitoneal hemorrhage, arteriovenous fistula, and dissection), and complications resulting from the use of contrast, which include allergic or adverse reaction from contrast, and contrast-induced nephropathy. Others include conduction disturbances, cerebrovascular accidents, cholesterol emboli, injury to intrathoracic great vessels and perforation of the cardiac chamber, myocardial infarction, iatrogenic coronary dissection, cardiac tamponade, and mortality [2].

## Conclusions

Coronary angiography remains the gold standard in the assessment of CAD. FFR and iFR are important physiological indexes for guiding revascularization in stable angina patients who have angiographically intermediate coronary stenoses.

Accurate knowledge and interpretation of coronary angiograms are necessary for successful and complete revascularization in patients who present with CAD. Valuable clinical information can still be obtained by aortography and ventriculography, although these investigative modalities have recently been overtaken by more recently developed modalities.

## References

[1] Loop FD (1987). Classics in thoracic surgery. F. Mason Sones, Jr., (1918-1985). Ann Thorac Surg.

[2] Tavakol M, Ashraf S, Brener SJ (2012). Risks and complications of coronary angiography: a comprehensive review. Glob J Health Sci.

[3] (2020). Cardiac Catheterization and Coronary Intervention. https://global.oup.com/academic/product/cardiac-catheterization-and-coronary-intervention-9780198705642?cc=us&lang=en&.

[4] Di Mario C, Sutaria N (2005). Coronary angiography in the angioplasty era: projections with a meaning. Heart.

[5] Mehr AZ (2022). Catheterization and angiography. Practical Cardiology: Principles and Approaches.

[6] Kern MJ, Klein AJ, Patel PM (2022). Coronary angiography and ventriculography, 7th ed., South East Asia Edition. Kern’s Cardiac Catheterization.

[7] Bhatia V, Arora P, Gupta A, Bhandari S, Kaul U (2019). “The moustache sign”: a common morphological characteristic in cardiovascular disease treatment. Heart Res Open J.

[8] Sachdev S, Omar B, Awan GM, Eyrich G (2018). Coronary angiography: basic views. Cardiofel Newslet.

[9] Selcuk E, Cevirme D, Bugra O (2020). Prognostic value of coronary dominance in patients undergoing elective coronary artery bypass surgery. Braz J Cardiovasc Surg.

[10] Lawton JS, Tamis-Holland JE, Bangalore S (2022). 2021 ACC/AHA/SCAI guideline for coronary artery revascularization: a report of the American College of Cardiology/American Heart Association Joint Committee on clinical practice guidelines. J Am Coll Cardiol.

[11] Natsumeda M, Nakazawa G, Murakami T (2015). Coronary angiographic characteristics that influence fractional flow reserve. Circ J.

[12] Park KH, Ahn Y, Koh YY (2019). Effectiveness and safety of zotarolimus-eluting stent (Resolute™ Integrity) in patients with diffuse long coronary artery disease. Korean Circ J.

[13] Zhou FF, Liu YH, Ge PC (2017). Coronary artery diameter is inversely associated with the severity of coronary lesions in patients undergoing coronary angiography. Cell Physiol Biochem.

[14] Ngaage DL, Hashmi I, Griffin S, Cowen ME, Cale AR, Guvendik L (2010). To graft or not to graft? Do coronary artery characteristics influence early outcomes of coronary artery bypass surgery? Analysis of coronary anastomoses of 5171 patients. J Thorac Cardiovasc Surg.

[15] Di Mario C, Werner GS, Sianos G (2007). European perspective in the recanalisation of chronic total occlusions (CTO): consensus document from the EuroCTO Club. EuroIntervention.

[16] Tonino PA, De Bruyne B, Pijls NH (2009). Fractional flow reserve versus angiography for guiding percutaneous coronary intervention. N Engl J Med.

[17] Götberg M, Christiansen EH, Gudmundsdottir IJ (2017). Instantaneous wave-free ratio versus fractional flow reserve to guide PCI. N Engl J Med.

[18] van Nunen LX, Zimmermann FM, Tonino PA (2015). Fractional flow reserve versus angiography for guidance of PCI in patients with multivessel coronary artery disease (FAME): 5-year follow-up of a randomised controlled trial. Lancet.

[19] (1985). The thrombolysis in myocardial infarction (TIMI) trial. Phase I findings. N Engl J Med.

[20] Madhavan MV, Tarigopula M, Mintz GS, Maehara A, Stone GW, Généreux P (2014). Coronary artery calcification: pathogenesis and prognostic implications. J Am Coll Cardiol.

[21] Yoshikai M, Kamohara K, Yunoki J (2002). Coronary artery bypass grafting to a calcified right coronary artery. Jpn J Thorac Cardiovasc Surg.

[22] Ellouze M, Bouchard D, Pham M, Noly PE, Perrault LP, Cartier R, Carrier M (2022). Coronary endarterectomy in patients with diffuse coronary artery disease: assessment of graft patency with computed tomography angiography. Can J Surg.

[23] Sternheim D, Power DA, Samtani R, Kini A, Fuster V, Sharma S (2021). Myocardial bridging: diagnosis, functional assessment, and management: JACC state-of-the-art review. J Am Coll Cardiol.

[24] Seiler C (2010). The human coronary collateral circulation. Eur J Clin Invest.

[25] Wainwright RJ, Maisey MN, Edwards AC, Sowton E (1980). Functional significance of coronary collateral circulation during dynamic exercise evaluated by thallium-201 myocardial scintigraphy. Br Heart J.

[26] Aboul-Enein F, Kar S, Hayes SW (2004). Influence of angiographic collateral circulation on myocardial perfusion in patients with chronic total occlusion of a single coronary artery and no prior myocardial infarction. J Nucl Med.

[27] Seiler C, Stoller M, Pitt B, Meier P (2013). The human coronary collateral circulation: development and clinical importance. Eur Heart J.

[28] Werner GS (2014). The role of coronary collaterals in chronic total occlusions. Curr Cardiol Rev.

[29] Gigliotti OS, Babb JD, Dieter RS (2015). Optimal use of left ventriculography at the time of cardiac catheterization: a consensus statement from the Society for Cardiovascular Angiography and Interventions. Catheter Cardiovasc Interv.

[30] Apostolakis EE, Baikoussis NG (2009). Methods of estimation of mitral valve regurgitation for the cardiac surgeon. J Cardiothorac Surg.

[31] Buckley RS, Kaul S, Jayaweera AR, Gimple LW, Powers ER, Dent JM (2000). Quantification of mitral regurgitation in the cardiac catheterization laboratory with contrast echocardiography. Am Heart J.

[32] Ahmed I, Hajouli S (2023). Left heart cardiac catheterization. StatPearls [Internet.

[33] Hunt D, Baxley WA, Kennedy JW, Judge TP, Williams JE, Dodge HT (1973). Quantitative evaluation of cineaortography in the assessment of aortic regurgitation. Am J Cardiol.

